# Henoch-Schönlein purpura in an older man presenting as rectal bleeding and IgA mesangioproliferative glomerulonephritis: a case report

**DOI:** 10.1186/1752-1947-5-364

**Published:** 2011-08-10

**Authors:** Wisit Cheungpasitporn, Teeranun Jirajariyavej, Charles B Howarth, Raquel M Rosen

**Affiliations:** 1 Department of Internal Medicine, Bassett Medical Center, Cooperstown, NY 13326, USA

## Abstract

**Introduction:**

Henoch-Schönlein purpura is the most common systemic vasculitis in children. Typical presentations are palpable purpura, abdominal pain, arthritis, and hematuria. This vasculitic syndrome can present as an uncommon cause of rectal bleeding in older patients. We report a case of an older man with Henoch-Schönlein purpura. He presented with rectal bleeding and acute kidney injury secondary to IgA mesangioproliferative glomerulonephritis.

**Case presentation:**

A 75-year-old Polish man with a history of diverticulosis presented with a five-day history of rectal bleeding. He had first noticed colicky left lower abdominal pain two months previously. At that time he was treated with a 10-day course of ciprofloxacin and metronidazole for possible diverticulitis. He subsequently presented with rectal bleeding to our emergency department. Physical examination revealed generalized palpable purpuric rash and tenderness on his left lower abdomen. Laboratory testing showed a mildly elevated serum creatinine of 1.3. Computed tomography of his abdomen revealed a diffusely edematous and thickened sigmoid colon. Flexible sigmoidoscopy showed severe petechiae throughout the colon. Colonic biopsy showed small vessel acute inflammation. Skin biopsy resulted in a diagnosis of leukocytoclastic vasculitis. Due to worsening kidney function, microscopic hematuria and new onset proteinuria, he underwent a kidney biopsy which demonstrated IgA mesangioproliferative glomerulonephritis. A diagnosis of Henoch-Schönlein purpura was made. Intravenous methylprednisolone was initially started and transitioned to prednisone tapering orally to complete six months of therapy. There was marked improvement of abdominal pain. Skin lesions gradually faded and gastrointestinal bleeding stopped. Acute kidney injury also improved.

**Conclusion:**

Henoch-Schönlein purpura, an uncommon vasculitic syndrome in older patients, can present with lower gastrointestinal bleeding, extensive skin lesions and renal involvement which responds well to systemic steroid therapy. A history of diverticulosis can mislead physicians to the diagnosis of diverticular bleeding which is more common in this age group. The clinical manifestations of the disease, including characteristic skin rash, abdominal pain, joint inflammation and renal involvement raised the suspicious of Henoch-Schönlein purpura.

## Introduction

Henoch-Schönlein purpura (HSP) is a predominantly pediatric vasculitic syndrome. Ninety percent of cases occur in the pediatric age group between the ages of 3 and 15 years. HSP occurs uncommonly in adults with an incidence rate of 0.1 to 1.2 per million in adults over 20-years old [1]. The classic tetrad of HSP includes palpable purpura without thrombocytopenia and coagulopathy, arthritis, abdominal pain and renal involvement. The extensive lower gastrointestinal hemorrhage due to colitis associated with vasculitis is an uncommon presentation of HSP and can be associated with an increased risk of renal involvement [2]. Conversely, colonic diverticular diseases such as diverticulitis and diverticular bleeding commonly present in older patients as left lower abdominal pain and rectal bleeding, respectively [3]. A documented history of diverticulosis in patients who present with gastrointestinal bleeding may mislead physicians to the wrong diagnosis and management. We report a case of Henoch-Schönlein purpura in an older man that presented as rectal bleeding and acute kidney injury secondary to IgA mesangioproliferative glomerulonephritis.

## Case Presentation

A 75-year-old Polish man with a history of kidney stones and colonic diverticulosis presented with bright red bleeding from his rectum for the previous five days to our emergency department. About two months prior, he had developed lower abdominal pain, left-sided more than right-sided. He was seen in Urgent Care and the diagnosis of urolithiasis was made as he had 6 to 10 red blood cells per high power field (RBCs/HPF) on urine analysis. He was referred to a urologist for further evaluation. Renal ultrasound was performed and showed benign-appearing bilateral renal cysts without renal stones or hydronephrosis. A cystoscopy was suggested, but not pursued. During the same period of time, he also noticed a generalized skin rash, more pronounced on his lower extremities. He was asymptomatic from the rash at that point with no itching or pain. No respiratory infections had occurred before the onset of the rash. He was seen by his family physician for follow up of his abdominal pain and was treated with a 10-day course of ciprofloxacin and metronidazole for possible diverticulitis as the patient had a known finding of diverticulosis on abdominal computed tomography in the past.

**Figure 1:**
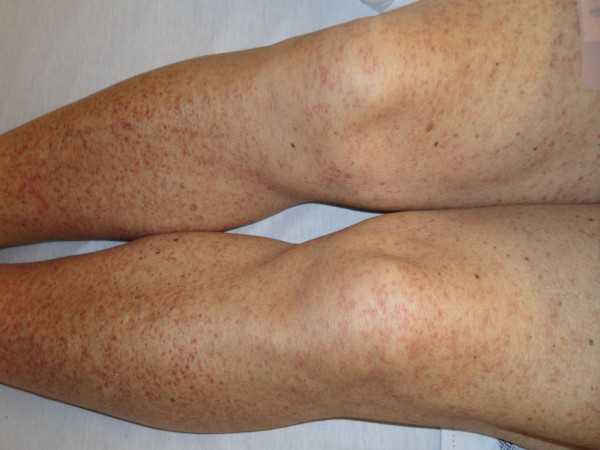
**Skin lesion of the patient on lower extremities**. Generalized palpable purpuric rash on both lower extremities.

**Figure 2:**
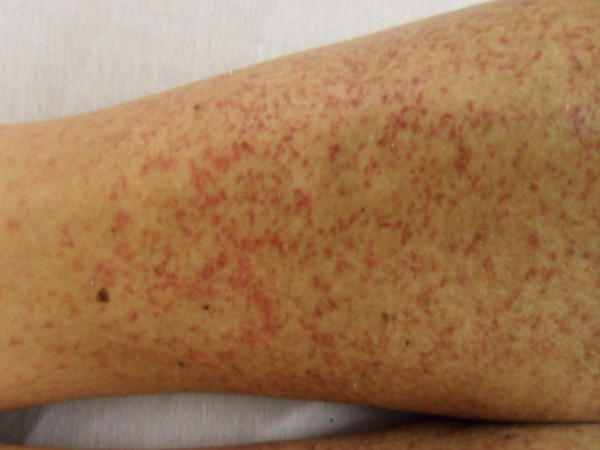
**Skin lesion of the patient on lower extremities**. Generalized palpable purpuric rash on lower extremities.

**Figure 3:**
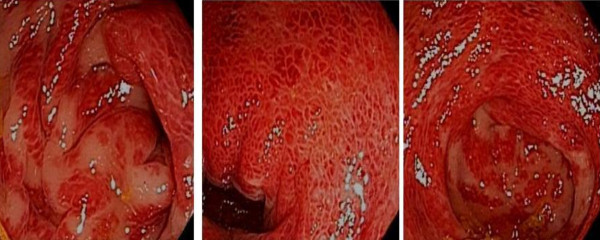
**Sigmoidoscopic findings of the patient**. This slide demonstrates vasculitic colitis in HSP.

He reported rectal bleeding and worsening left lower abdominal pain for five days prior to presenting to the emergency department for evaluation. He had had swollen bilateral proximal interphalangeal (PIP) joints of his hands in the past two years; however, there was no currently active joint pain. He denied having Raynaud's disease, sun sensitivity, pleurisy, urethritis, oral aphthae, alopecia, or acute eye problems. He also denied recent history of non-steroidal anti-inflammatory drugs and angiotensin-converting enzyme inhibitors use, food allergies, and vaccinations or insect bites. On physical examination, there was a generalized, palpable, purpuric rash on his trunk and both extremities, more pronounced on his lower extremities and buttocks (Figures 1 and 2). Abdominal examination showed mild tenderness of his left lower abdomen without guarding or rebound. There was bilateral pedal edema without significant joint swelling. Laboratory testing showed a mildly elevated serum creatinine of 1.3. Urine analysis was remarkable for microscopic hematuria; dysmorphic RBCs 20 to 25, and new onset proteinuria; urine protein-to-creatinine ratio was 1.53. The C-reactive protein was slightly elevated at 1.3. Additional blood tests included anti-nuclear antibody (ANA), cryoglobulins, hepatitis B and C antibodies, anti-double stranded DNA antibodies, complement levels, serum protein electrophoresis, and myeloperoxidase and PR3 antibodies that were negative. Abdominal computed tomography with contrast showed a diffusely edematous and thickened sigmoid colon and probably the rectum with surrounding inflammation. The possibilities of infectious colitis, ischemic colitis, and vasculitis such as small vessel and drug-induced vasculitis were raised. Emergency flexible sigmoidoscopy was performed and showed severe petechiae starting just above the anal verge, throughout the examined part of the colon, and much more pronounced in the rectal area (Figure 3). Colonic biopsy demonstrated small vessel, acute inflammation in colonic mucosa with superficial hemorrhage and patchy, acute cryptitis. Skin biopsy revealed leukocytoclastic vasculitis involving the small vessels. A direct immunofluorescent technique showed rare colloid bodies with antibodies to IgG and IgA, trace granular basement membrane staining with antibodies to C3 and trace basement membrane staining with antibodies to IgM. With these clinical and laboratory results he was diagnosed with a case of adult onset HSP and was initially treated with intravenous methylprednisolone 125 mg every 8 hours for one day and intravenous isotonic fluids. There was marked improvement of abdominal pain. Skin lesions gradually faded away and rectal bleeding resolved. After nephrology evaluation of acute kidney injury, along with proteinuria and hematuria, he underwent a kidney biopsy which demonstrated IgA mesangioproliferative glomerulonephritis with less than 50% crescents. Oral prednisone tapering was continued to complete the six months of steroid therapy. Acute kidney injury and proteinuria were markedly improved after one month of treatment. He was doing well without another episode of rectal bleeding.

## Discussion

HSP, a vasculitic syndrome characterized by skin rash, abdominal colic, joint pain and glomerulonephritis, was first described in 1801 by Dr. William Heberden [4]. The syndrome is mainly a disease of early childhood with most cases presenting by 10 years of age. It is uncommon in adults over the age of 20. Men are affected more than women with a ratio of 1.2:1 to 1.8:1 [5]. A recent history of respiratory tract infection is reported in 90% of cases. Other precipitating factors, reported in the adult onset of HSP, include medications (non-steroidal anti-inflammatory drugs, angiotensin-converting enzyme inhibitors, and antibiotics such as vancomycin and cefuroxime), food allergies, vaccinations, and insect bites.

The clinical manifestations of HSP may develop over the course of days to weeks and may vary in their order of presentation; however, renal involvement usually presents late. The purpuric skin lesions are typically located on the lower extremities but may also be seen on the hands, arms, trunk, and buttocks.

Gastrointestinal disease occurs in up to 70% of patients varying from colicky abdominal pain, nausea and vomiting to intestinal hemorrhage, intussusceptions, pancreatitis and hydrops of the gall bladder. More than 30% of patients experience diffuse pain described as 'bowel angina' typically occurring after meals and accompanied by bloody diarrhea. Renal involvement is usually noted within a few days to one month after the onset of systemic symptoms. Renal manifestations occur more commonly and tend to be more severe in adults including end-stage renal disease [6]. Urinary abnormalities are present in 25% to 50% of patients. Hematuria is the most common symptom and the earliest sign of renal involvement. Although early studies suggested that renal involvement could not be predicted from the severity of extra-renal involvement, a recent study showed that a recent infectious history, pyrexia, spread of purpura to the trunk, and biological markers of inflammation were predictive factors for renal involvement [7]. The risk of renal involvement is also increased when HSP patients present with bloody stools [2] as in our patient.

Joint involvement occurs in 60% to 84% of cases and generally affects ankles and knees. In adults, involvement of the small joints is more common [8]. Our patient did not experience active joint symptoms which is an atypical presentation.

The diagnosis of HSP is based on clinical signs and symptoms. Laboratory studies generally show a mild leukocytosis, a normal platelet count, and occasionally eosinophilia. Serum complement components are normal. IgA levels are elevated in about one-half of patients. In patients with unusual presentations, a biopsy of an affected organ (for example, skin or kidney) that demonstrates leukocytoclastic vasculitis with a predominance of IgA deposition confirms the diagnosis of HSP.

A kidney biopsy can be done to establish the diagnosis, but this invasive procedure is generally reserved for patients in whom the diagnosis is uncertain or who have more severe renal involvement such as marked proteinuria and/or impaired renal function during the acute episode. The percentage of glomeruli showing crescents is the most important prognostic finding.

The long term prognosis of HSP is almost entirely determined by the behavior of the nephritis. The short term outcome of renal disease in HSP is favorable in most patients, with complete recovery reported in 94% of children and 89% of adults [9]. Recurrence of HSP is common, occurring in up to one-third of patients and more likely in patients with renal involvement. Among adults, the reported rates of end-stage renal disease range from 10% to 30% at 15 years.

There is no specific treatment for HSP. The majority of cases are mild and need only supportive measures. Although there is evidence suggesting that corticosteroids enhance the rate of resolution of the arthritis and abdominal pain, they do not seem to prevent recurrence of disease. Aggressive therapy with corticosteroids or cyclophosphamide has not been proven to be beneficial in reversing the renal disease except among patients with crescentic nephritis [10]. However, some experts recommend a six-month course of corticosteroids for patients with the nephrotic syndrome and those with a reduced glomerular filtration rate. Renal transplantation can be performed in those patients who progress to end-stage renal disease.

Our patient underwent a kidney biopsy because of marked proteinuria and acute kidney injury. The biopsy showed IgA mesangioproliferative glomerulonephritis with less than 50% crescents indicating a good prognosis. He was initially treated with intravenous methylprednisolone and was transitioned to prednisone tapering orally to complete the six-months of steroid therapy. There was marked improvement of abdominal pain and gastrointestinal bleeding. Skin lesions gradually faded. Acute kidney injury and proteinuria also improved.

## Conclusion

HSP is a vasculitic syndrome that can present with extensive skin lesions, lower gastrointestinal bleeding and renal involvement even in very old patients and responds well to systemic steroid therapy. A history of diverticulosis can mislead physicians to a diagnosis of diverticular bleeding which is more common in this age group. Physicians should be suspicious of HSP in patients who present with clinical manifestations of the disease comprising characteristic skin rash, abdominal colic, joint pain and renal involvement.

## Abbreviations

ANA: anti-nuclear antibody; Anti PR-3 Ab: anti-proteinase-3 antibodies; HSP: Henoch-Schönlein purpura; Ig: Immunoglobulin; PIP: proximal interphalangeal; RBC/HPF: red blood cells per high power field.

## Consent

Written informed consent was obtained from the patient for publication of this case report and any accompanying images. A copy of the written consent is available for review by the Editor-in-Chief of this journal.

## Competing interests

The authors declare that they have no competing interests.

## Authors' contributions

WC, CBH, and RMR were involved in the diagnosis and treatment of the patient. WC and TJ drafted the manuscript. CBH and RMR revised the manuscript. All authors read and approved the final manuscript.
